# Four weeks of isometric handgrip training results in lower awake ambulatory blood pressure compared with control: a randomized clinical trial

**DOI:** 10.1111/cpf.70102

**Published:** 2026-10-25

**Authors:** Patrícia C. Oliveira, Gustavo Waclawovsky, Alexandre M. Lehnen

**Affiliations:** ^1^ Institute of Cardiology of Rio Grande do Sul/University Foundation of Cardiology Porto Alegre Rio Grande do Sul Brazil

**Keywords:** antihypertensive therapy, cardiovascular physiology, exercise therapy, handgrip, knee extension, muscle mass

## Abstract

We compared the effects of upper‐limb (handgrip) and lower‐limb (knee extension) isometric training on ambulatory blood pressure in individuals with controlled hypertension. In this randomized controlled trial, 67 adults receiving antihypertensive medication were allocated to a control group (CG, *n* = 22), small muscle mass isometric training (SMM, handgrip; *n* = 22), or large muscle mass isometric training (LMM, knee extension; *n* = 23). Participants completed 12 supervised sessions over 4 weeks, with the exercise protocols performed at 30% of upper‐limb MVC or lower‐limb maximal isometric load, respectively. Ambulatory blood pressure outcomes were compared using analysis of covariance (ANCOVA), adjusting for the corresponding baseline office blood pressure. Hourly 24‐h profiles were analyzed using generalized estimating equations with Bonferroni‐adjusted post hoc tests. Significant between‐group differences were observed only for awake ambulatory blood pressure. Compared with CG, SMM showed lower awake systolic blood pressure (121.8 ± 3.1 vs. 124.2 ± 2.6 mmHg; *p* = 0.017) and awake diastolic blood pressure (76.3 ± 3.6 vs. 80.8 ± 4.2 mmHg; *p* = 0.009). No significant differences were observed between LMM and CG, between the two exercise groups, or for 24‐h and sleep blood pressure. These findings indicate that, after 4 weeks of supervised isometric training, modest between‐group differences in awake ambulatory blood pressure were observed only for handgrip training compared with the control group. Greater muscle mass recruitment did not result in superior antihypertensive effects, suggesting that the chronic blood pressure response to isometric exercise is not determined solely by the amount of muscle mass involved.

**Trial registration:** “ClinicalTrials.gov,” “NCT07147595.”

## 1 INTRODUCTION

Noncommunicable chronic diseases, such as systemic arterial hypertension (SAH), remain among the leading challenges to global public health because they are strongly associated with the principal causes of mortality worldwide (WHO, 2022). SAH is the most important modifiable risk factor for adverse cardiovascular outcomes, accounting for approximately 13% of cardiovascular events, followed by smoking (9%), elevated blood glucose (6%), physical inactivity (6%), and excess body weight (5%) (Malta et al., 2011).

Lifestyle modification, particularly regular physical activity, is a cornerstone of SAH management. In 2022, approximately 31% of adults worldwide did not meet the minimum recommended levels of physical activity (150 min per week of moderate‐intensity activity, 75 min of vigorous‐intensity activity, or an equivalent combination) (Strain et al., 2024). In Brazil, nearly half of the adult population did not meet this recommendation in 2019 (IBGE, 2020).

Regular aerobic exercise is widely recognized as the first‐line nonpharmacological intervention for SAH, whereas dynamic resistance training is recommended as an adjunctive therapy (Brandao et al., 2025; McEvoy et al., 2024; Writing Committee et al., 2025). More recently, isometric exercise has emerged as a promising antihypertensive strategy. Accordingly, the American Heart Association (AHA) has incorporated isometric exercise into its recommendations for blood pressure management. However, the current evidence remains heterogeneous, and uncertainty persists regarding both its efficacy and the magnitude of its antihypertensive effects. While several studies have demonstrated clinically meaningful reductions in blood pressure following isometric training (Carlson et al., 2014; Carlson et al., 2016; Inder et al., 2016; Oliveira et al., 2025), others have reported no significant benefits (Ash et al., 2017; Olher et al., 2013; Silva et al., 2018).

Several factors may explain these inconsistent findings, including differences in baseline blood pressure, exercise protocols (e.g., unilateral vs. bilateral contractions), training volume, and contraction characteristics. An additional factor that has received comparatively little attention is the amount of muscle mass involved during exercise. Most available evidence is derived from isometric handgrip protocols, which primarily involve a relatively small muscle mass (Nemoto et al., 2021; Punia & Kulandaivelan, 2020; Seidel et al., 2021). In contrast, studies involving larger muscle groups, such as the quadriceps, have predominantly included normotensive participants (Baross et al., 2012; Baross et al., 2013; Edwards, Jalaludeen, et al., 2023; Wiles et al., 2010; Wiles et al., 2018). Moreover, ambulatory blood pressure responses have been evaluated in relatively few studies, despite their greater clinical relevance than office blood pressure measurements. Evidence regarding the effects of lower‐limb isometric exercise on 24‐h ambulatory blood pressure monitoring (ABPM) in individuals with hypertension also remains limited (Taylor et al., 2019).

Therefore, we conducted a randomized clinical trial to compare the effects of upper‐limb (handgrip) and lower‐limb (knee extension) isometric training on 24‐h ambulatory blood pressure in individuals with controlled hypertension. We hypothesized that both isometric exercise protocols would reduce ambulatory blood pressure compared with the control group, with lower‐limb isometric training producing greater antihypertensive effects than handgrip exercise.

## 2 METHODS

This was a randomized, parallel‐group, assessor‐blinded clinical trial conducted in accordance with the CONSORT Statement and the Declaration of Helsinki. The protocol was approved by the Research Ethics Committee of the Institute of Cardiology of Rio Grande do Sul/University Foundation of Cardiology (ICFUC) under approval number 5.373.371 (April 27, 2022). Data were collected between September 2023 and February 2025. The trial was registered at ClinicalTrials.gov (NCT07147595). All participants provided written informed consent before enrolment.

### 2.1 Participants and randomization

Participants aged 40–70 years, with a diagnosis of hypertension for at least 1 year and receiving antihypertensive medication, were recruited through the electronic medical records of the ICFUC cardiovascular rehabilitation facility. Exclusion criteria included a history of myocardial infarction, heart failure, documented atherosclerosis, diabetes mellitus, renal insufficiency, body mass index (BMI) ≥35 kg/m^2^, or upper and/or lower limb injuries that could prevent exercise execution. Also, office blood pressure was measured at baseline as part of the screening procedures to determine eligibility according to the predefined inclusion criteria and was subsequently included as a covariate in the statistical analysis.

Sample size was calculated using the online PSS Health tool. Considering three groups, two time points, and the group‐by‐time interaction, with 80% power, a significance level of 5%, and expected mean changes of 5.8 ± 8.0 mmHg within groups and 7.2 ± 7.1 mmHg between groups, based on a previous trial (Ash et al., 2017), the required sample size was estimated at 66 participants (22 per group).

After screening and enrolment, participants were randomly assigned in a 1:1:1 ratio to one of three groups—control group (CG), small muscle mass (SMM), or large muscle mass (LMM)—using simple randomization generated through Randomization.com. The allocation sequence was generated by a researcher who was not involved in participant assessment or intervention delivery. Due to the nature of the interventions, participants and exercise supervisors could not be blinded to group allocation. However, outcome assessments were conducted by evaluators who were blinded to group allocation.

### 2.2 Study visits and data collection

All assessments and intervention or control sessions were conducted in the afternoon to minimize circadian variability. Visit 2 was performed 24–48 h after Visit 1. Participants were instructed to abstain from physical exercise, caffeine, and alcohol for 24 h before each visit. Procedures were standardized across groups whenever applicable to minimize potential measurement and procedural variability.

Importantly, throughout the study, participants were instructed to maintain their usual daily routines, including habitual physical activity, dietary habits, sleep patterns, social activities, and prescribed medication regimens, without introducing any changes.

*Visit 1:* written informed consent, assessment of habitual physical activity using the long version of the International Physical Activity Questionnaire (IPAQ), and collection of blood tests for biochemical characterization;

*Visit 2:* anthropometric measurements (body weight, height, and waist circumference) and determination of maximal voluntary contraction (MVC) for the upper limbs and maximal isometric load for the lower limbs.

*Intervention or control visits (12 sessions):* verification of antihypertensive medication use and dietary intake during the preceding 24 h, followed by clinic blood pressure measurement. Exercise (handgrip or knee extension) or control procedures were then performed. Clinic blood pressure was reassessed for up to 15 min after each session before participants were discharged. All groups completed 3 weekly sessions for 4 weeks. ABPM was initiated 24 h after the final session and continued for 24 h. Awake and sleep periods were predefined in the ABPM software as 07:00 a.m.–11:00 p.m. and 11:00 p.m.–07:00 a.m., respectively.

Baseline ABPM was initially planned as part of the study protocol. However, during the initial phase of the study, four volunteers underwent baseline ABPM and subsequently withdrew from participation. Given these early withdrawals and concerns regarding participant burden and retention, the assessment schedule was modified to include a single ABPM measurement after the 4‐week intervention. This decision was also informed by previous research evaluating ambulatory blood pressure responses following exercise (Ferrari et al., 2017). Baseline office blood pressure, obtained during screening, was retained as a pre‐intervention clinical measure and subsequently used as a covariate in the statistical analysis.

### 2.3 Anthropometric assessment

Body weight and height were measured with participants wearing light clothing and barefoot, using a mechanical scale with stadiometer (Welmy W200, São Paulo, Brazil). Waist circumference was measured at the narrowest point between the ribs and iliac crest using a flexible, nonelastic tape (Sanny, São Paulo, Brazil), without compressing the skin. When the narrowest point was not identifiable, the measurement was taken 2 cm above the umbilicus.

### 2.4 Maximal voluntary contraction (MVC)

Upper limbs: MVC was assessed using a hydraulic handgrip dynamometer (Jamar), set at handle position II, in accordance with the recommendations of the American Society of Hand Therapists. Tests were performed bilaterally with participants seated, feet supported on the floor, shoulder in neutral rotation, elbow flexed at 90°, forearm in mid‐pronation, and wrist in a neutral position. Three trials were performed for each arm, separated by 1‐min intervals, and the highest value was recorded.

Lower limbs: maximal isometric load was assessed using adjustable ankle weights targeting the knee extensor muscles. Participants were seated with their feet supported on the floor. Evaluators assisted with initial positioning to ensure that each trial began under isometric conditions. Participants were instructed to sustain the knee extension for at least 3 s. They were allowed to hold the sides of the chair with both hands to maintain trunk stability but were instructed to avoid excessive upper‐limb force. Three trials with progressively increasing loads were performed, separated by 1‐min intervals. The highest load (kg) that could be fully sustained for at least 3 s was defined as the maximal isometric load and was operationally considered the lower‐limb MVC.

### 2.5 Blood pressure assessment

For clinic blood pressure measurement, participants remained seated quietly for 10 min with back support and feet supported on the floor. Blood pressure was measured in each arm using a semi‐automated device (OMRON® 705CP, São Paulo, Brazil), and the arm with the higher reading was used for subsequent measurements.

For 24‐h ABPM, an oscillometric device (Dyna‐MAPA, São Paulo, Brazil) was placed on the left arm and programmed to obtain three readings per hour during waking hours and two readings per hour during sleep. ABPM recordings were considered valid when they included at least one valid reading per hour and ≥85% valid readings over the 24‐h monitoring period.

### 2.6 Intervention protocol

The SMM group performed four sets of 2‐min unilateral isometric handgrip contractions at 30% MVC using a handgrip dynamometer (Jamar), alternating sides between sets. The LMM group performed four sets of 2‐min unilateral isometric knee extensions at 30% MVC using adjustable ankle weights, also alternating sides between sets. Both exercise groups rested for 1 min between sets. The CG underwent the same study procedures except for exercise; during each intervention session, participants remained seated for 25 min to match the duration of the exercise sessions.

### 2.7 Statistical analysis

Normality and homogeneity of variances were assessed using the Shapiro–Wilk and Levene tests, respectively. Data are presented as mean ± standard deviation or estimated marginal means with 95% confidence intervals (95% CI), as appropriate. Baseline characteristics were compared among groups using one‐way analysis of variance (ANOVA) with Bonferroni‐adjusted post hoc comparisons, except for habitual physical activity (IPAq), for which Tukey's post hoc test was applied.

Between‐group differences in ABPM outcomes were evaluated using analysis of covariance (ANCOVA). For each ABPM outcome, the corresponding baseline office blood pressure (systolic or diastolic) was included as a covariate. Before fitting the final ANCOVA models, Pearson correlation analysis was performed to assess the relationship between baseline office blood pressure and the corresponding ABPM outcome. The assumption of homogeneity of regression slopes was assessed by testing the interaction between group and the respective baseline office blood pressure. As the interaction terms were not statistically significant for any outcome, they were removed from the final models. Adjusted between‐group comparisons were performed using estimated marginal means with Bonferroni correction for multiple comparisons. Effect sizes were expressed as partial eta squared (*η* ^2^ *p*).

Hourly 24‐h ambulatory blood pressure profiles were analyzed using generalized estimating equations (GEE), including group, time, and the group‐by‐time interaction as factors, with a normal probability distribution, identity link function, and an exchangeable working correlation structure. Bonferroni‐adjusted pairwise comparisons were performed when appropriate.

All statistical analyses were conducted using a per‐protocol approach, including participants who completed the assigned intervention or control protocol and had available outcome data. Also, statistical analyses were performed using IBM SPSS Statistics version 23.0 (IBM Corp.), and statistical significance was set at *p* < 0.05.

## 3 RESULTS

Among the individuals screened, five did not meet the inclusion criteria, four declined to undergo 24‐h ABPM, and eight did not proceed for commuting difficulties or personal reasons (Figure 1). The final study sample comprised 67 participants (22 men and 45 women: CG, 8 men and 14 women; SMM, 7 men and 15 women; LMM, 7 men and 16 women), with a mean age of 56.3 ± 6.4 years and a BMI of 29.8 ± 3.6 kg/m^2^.

**FIGURE 1:**
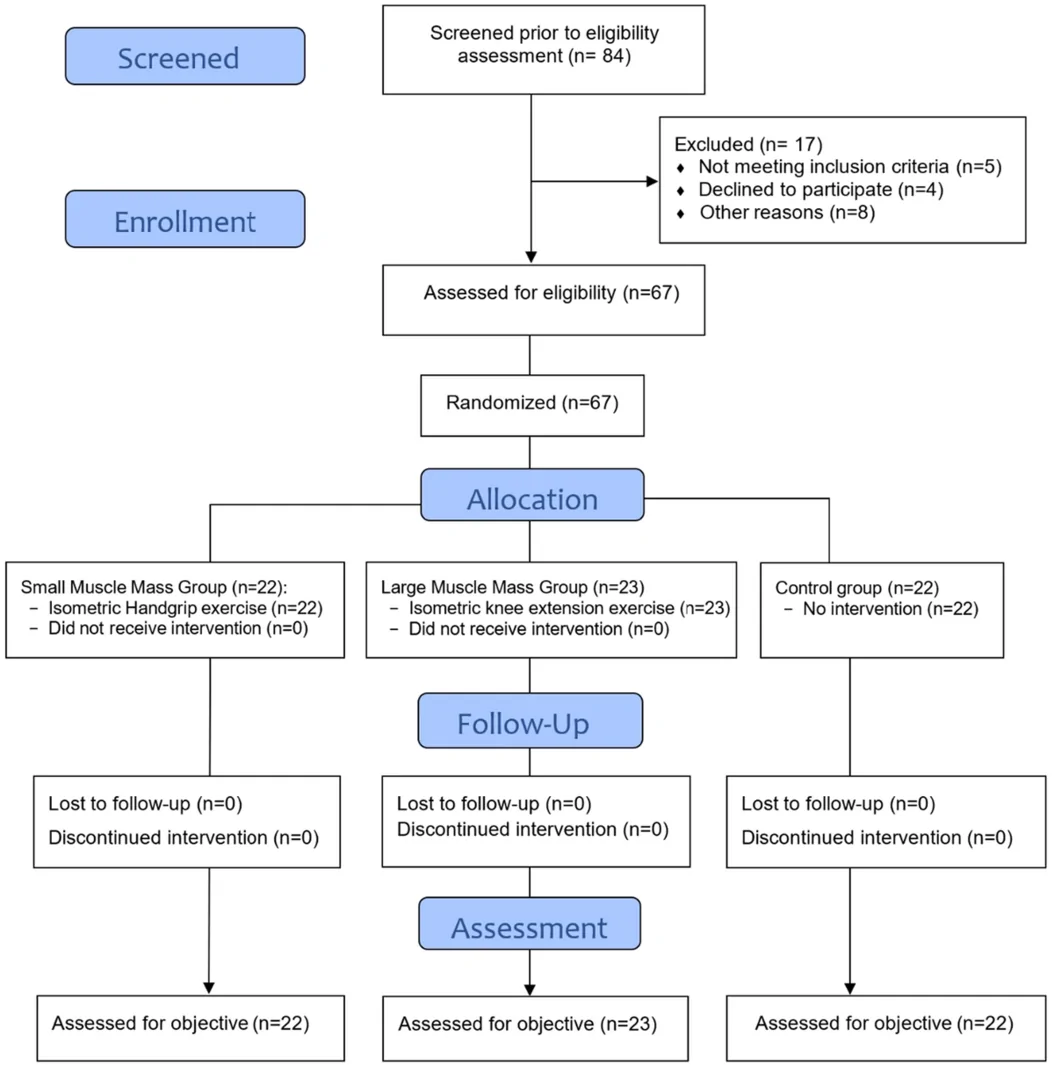
Study design. ABPM, 24‐h ambulatory blood pressure monitoring. BP, blood pressure. Small Muscle Mass (SMM) group underwent isometric handgrip exercise; Large Muscle Mass (LMM) group underwent isometric knee extension exercise; Control Group (CG) with no exercise.

Baseline characteristics are presented in Table 1. The groups were comparable regarding age, BMI, waist circumference, fasting blood glucose, glycated haemoglobin, and lipid profile. Height was lower in the LMM group than in both the CG and SMM groups, while body weight was lower in the LMM group than in the CG. BMI did not differ among the groups. Habitual physical activity differed significantly among groups (*p* = 0.034), with higher MET‐hours per week observed only in the LMM group compared with the CG (*p* = 0.031).

**TABLE 1:** Baseline characteristics of the groups studied.

	CG (*n* = 22) Mean ± SD (95% CI)	SMM (*n* = 22) Mean ± SD (95% CI)	LMM (*n* = 23) Mean ± SD (95% CI)	*p*‐value
Age (years)	56 ± 7 (53; 59)	59 ± 5 (56; 61)	54 ± 6 (52; 57)	0.060
Height (m)	1.70 ± 0.08 (1.67; 1.74)	1.71 ± 0.07 (1.67; 1.74)	1.64 ± 0.09 (1.60; 1.68)*, † ^,^ †	0.008
Body weight (kg)	88.4 ± 9.4 (84.2; 92.6)	83.8 ± 12.7 (78.1; 89.4)	76.9 ± 11.3 (72.0; 81.8)*	0.004
BMI (kg/m^2^)	31.1 ± 4.2 (29.3; 33.0)	28.6 ± 3.7 (27.0; 30.2)	29.8 ± 4.2 (28.0; 31.7)	0.178
Waist circumference (cm)	87.6 ± 6.2 (84.8; 90.3)	87.1 ± 7.3 (83.9; 90.4)	84.6 ± 9.4 (80.5; 88.6)	0.375
Fasting blood glucose (mg/dL)	93.2 ± 7.7 (89.9; 96.6)	98.0 ± 5.3 (95.7; 100.4)	96.1 ± 7.0 (93.1; 99.1)	0.065
HbA1c (%)	5.4 ± 0.3 (5.2; 5.5)	5.3 ± 0.4 (5.1; 5.5)	5.1 ± 0.5 (4.9; 5.3)	0.156
Total cholesterol (mg/dL)	185.4 ± 8.8 (181.5; 189.2)	198.7 ± 22.3 (188.9; 208.6)	194.1 ± 24.7 (183.4; 204.8)	0.086
HDL (mg/dL)	55.2 ± 6.5 (52.4; 58.1)	55.1 ± 6.5 (52.4; 58.1)	55.4 ± 7.1 (52.3; 58.5)	0.991
LDL (mg/dL)	78.2 ± 9.4 (74.0; 82.3)	81.6 ± 9.4 (78.7; 84.6)	83.2 ± 6.6 (80.3; 86.0)	0.085
Triglycerides (mg/dL)	209.1 ± 31.4 (195.2; 223.0)	208.2 ± 26.1 (196.6; 219.8)	213.3 ± 33.7 (198.8; 227.9)	0.835
Physical activity (MET‐h/week)	6.3 ± 2.3 (5.3; 7.3)	6.8 ± 2.1 (5.9; 7.8)	7.9 ± 1.7 (7.2; 8.7)*	0.034

*Note*: Baseline differences were analyzed using one‐way ANOVA followed by Bonferroni‐adjusted post hoc tests, except for habitual physical activity, which was analyzed using Tukey's post hoc test.

Abbreviations: BMI, body mass index; CG, control group; HbA1c, glycated haemoglobin; HDL, high‐density lipoprotein; LDL, low‐density lipoprotein; LMM, large muscle mass (isometric knee extension training); SMM, small muscle mass (isometric handgrip training).

* *p* < 0.05 versus CG.

† *p* < 0.05 versus SMM.

Table 2 summarizes the medications used by the participants. Overall, 43.3% of participants were taking up to two antihypertensive medications, whereas 56.7% were receiving three or more drugs. No statistically significant between‐group differences were observed in the distribution of medication classes.

**TABLE 2:** Medications used by the study participants.

	Total (*n* = 67)	CG (*n* = 22)	SMM (*n* = 22)	LMM (*n* = 23)	*p*‐valour
Angiotensin‐receptor blockers	37 (55%)	13	14	10	0.360
ACE inhibitors	19 (28%)	6	5	8	0.663
Statins	39 (58%)	14	9	16	0.123
Diuretics	32 (48%)	10	10	12	0.872
Betablockers	17 (25%)	5	7	5	0.696
Antiplatelets	8 (12%)	3	3	2	0.839
Calcium channel blockers	24 (36%)	7	9	8	0.814

*Note*: Between‐group differences in medication use were assessed using Pearson's chi‐square test or Fisher's exact test, as appropriate. (*p* < 0.05).

Abbreviations: ACE, angiotensin‐converting enzyme; CG, control group; LMM, large muscle mass (isometric knee extension exercise); SMM, small muscle mass (isometric handgrip exercise).

Table 3 presents upper‐limb MVC and lower‐limb maximal isometric load at baseline and after the intervention. At baseline, handgrip MVC was similar between the CG and SMM groups for both hands. In contrast, the LMM group exhibited greater maximal isometric loads than the CG for both the left (33%) and right (31%) legs.

**TABLE 3:** Upper‐limb maximal voluntary contraction and lower‐limb maximal isometric load.

	(A) Isometric Handgrip Right‐hand MVC (kgf)		(B) Isometric Handgrip Left‐hand MVC (kgf)	
	Baseline Mean ± SD (95% CI)	Post training Mean ± SD (95% CI)	Baseline Mean ± SD (95% CI)	Post training Mean ± SD (95% CI)
**CG (*n*** = **22)**	24.1 ± 6.4 (21.4; 26.8)	24.3 ± 6.2 (21.7; 26.9)	23.5 ± 6.4 (20.7; 26.4)	24.0 ± 6.8 (20.4; 26.5)*
**SMM (*n*** = **22)**	21.2 ± 6.3 (18.6; 23.8)	23.5 ± 6.8 (20.7; 26.2)†	20.5 ± 6.7 (17.6; 23.5)	23.2 ± 7.0 (20.1; 26.4)*

	(C) Maximal Isometric Knee Extension Load – Right (kg)		(D) Maximal Isometric Knee Extension Load – Left (kg)	
	Baseline Mean ± SD (95% CI)	Post training Mean ± SD (95% CI)	Baseline Mean ± SD (95% CI)	Post training Mean ± SD (95% CI)
**CG (*n*** = **22)**	18.4 ± 2.5 (17.3; 19.4)	18.5 ± 2.9 (17.2; 19.6)	17.3 ± 2.5 (16.3; 18.3)	17.1 ± 2.7 (16.0; 18.2)
**LMM (*n*** = **23)**	24.5 ± 7.2 (21.4; 27.6)†	27.1 ± 7.6 (23.8; 30.4)† ^,^ *	22.7 ± 7.1 (19.6; 25.8)†	25.1 ± 8.0 (21.5; 28.6)† ^,^ *

*Note*: Differences were tested using GEE, including group, time, and the group × time interaction, as following: (A) *p*‐value (interaction) = 0.023; (B) *p*‐value (interaction) <0.001; (C) *p*‐value (interaction) <0.001; (D) *p*‐value (interaction) <0.001. Later, a Bonferroni post hoc test was performed.

Abbreviations: CG, control group; kgf, kilogram‐force; LMM, large muscle mass (isometric knee extension exercise); MVC, maximum voluntary contraction; SMM, small muscle mass (isometric handgrip exercise).

* *p* < 0.05 versus baseline in the same group.

† *p* < 0.05 versus CG in the same time.

Office and ambulatory blood pressure outcomes following the intervention are presented in Table 4. After adjustment for the corresponding baseline office blood pressure using ANCOVA, significant between‐group differences were observed for awake systolic blood pressure (*p* = 0.015) and awake diastolic blood pressure (*p* = 0.012). Bonferroni‐adjusted pairwise comparisons demonstrated that the SMM group exhibited lower awake systolic blood pressure than the CG (121.8 ± 3.1 vs. 124.2 ± 2.6 mmHg, *p* = 0.017), as well as lower awake diastolic blood pressure (76.3 ± 3.6 vs. 80.8 ± 4.2 mmHg, *p* = 0.009). No significant differences were observed between the LMM and CG or between the two exercise groups. Likewise, no significant between‐group differences were detected for 24‐h systolic blood pressure (*p* = 0.126), 24‐h diastolic blood pressure (*p* = 0.203), sleep systolic blood pressure (*p* = 0.928), or sleep diastolic blood pressure (*p* = 0.858).

**TABLE 4:** Office and ambulatory blood pressure outcomes according to study group.

	CG (*n* = 22) Adjusted mean (95% CI)	SMM (*n* = 22) Adjusted mean (95% CI)	LMM (*n* = 23) Adjusted mean (95% CI)	*p*‐value
Office SBP (mmHg)	131.6 ± 8.5 (127.8; 135.4)	129.6 ± 7.8 (126.2; 133.1)	126.8 ± 7.5 (123.6; 130.1)	0.138
Office DBP (mmHg)	84.5 ± 6.8 (81.5; 87.5)	80.5 ± 6.5 (77.6; 83.4)	81.3 ± 6.8 (78.3; 84.2)	0.117
24‐h SBP (mmHg)	120.3 ± 3.1 (119.0; 121.7)	118.6 ± 2.8 (117.4; 119.8)	118.2 ± 2.7 (117.1; 119.4)	0.126
24‐h DBP (mmHg)	77.1 ± 5.5 (74.6; 79.5)	73.3 ± 4.3 (71.4; 75.2)	74.6 ± 4.6 (72.6; 76.5)	0.203
Awake SBP (mmHg)	124.2 ± 2.6 (123.0; 125.4)	121.8 ± 3.1 (120.5; 123.2)*	121.6 ± 2.8 (120.4; 122.8)	0.015
Sleep SBP (mmHg)	112.5 ± 5.6 (110.0; 115.0)	112.1 ± 2.7 (110.9; 113.3)	111.5 ± 3.3 (110.1; 113.0)	0.928
Awake DBP (mmHg)	80.8 ± 4.2 (78.9; 82.6)	76.3 ± 3.6 (74.7; 78.0)*	78.4 ± 3.9 (76.7; 80.0)	0.012
Sleep DBP (mmHg)	69.9 ± 9.0 (65.6; 73.9)	67.2 ± 6.8 (64.2; 70.2)	66.9 ± 7.5 (63.7; 70.2)	0.858

*Note*: Office blood pressure was measured at baseline and compared between groups using one‐way ANOVA. Between‐group comparisons for ambulatory blood pressure monitoring (ABPM) outcomes were performed using analysis of covariance (ANCOVA), with the corresponding baseline office systolic or diastolic blood pressure entered as the covariate.

* *p* < 0.05 versus CG according to Bonferroni‐adjusted pairwise comparisons following ANCOVA.

Hourly ABPM profiles over the 24‐h period following the intervention are shown in Figure 2. GEE analysis showed significant effects of group, time, and group‐by‐time interaction for both systolic and diastolic blood pressure (all *p* < 0.05). Bonferroni‐adjusted pairwise comparisons showed lower systolic blood pressure in both exercise groups than in the CG at selected time points, although these differences were not consistently observed throughout the 24‐h monitoring period. For diastolic blood pressure, significant pairwise differences relative to the CG were observed at a greater number of time points in the SMM group than in the LMM group. No adverse events or unintended events were reported in any of the three study groups during the intervention period.

**FIGURE 2:**
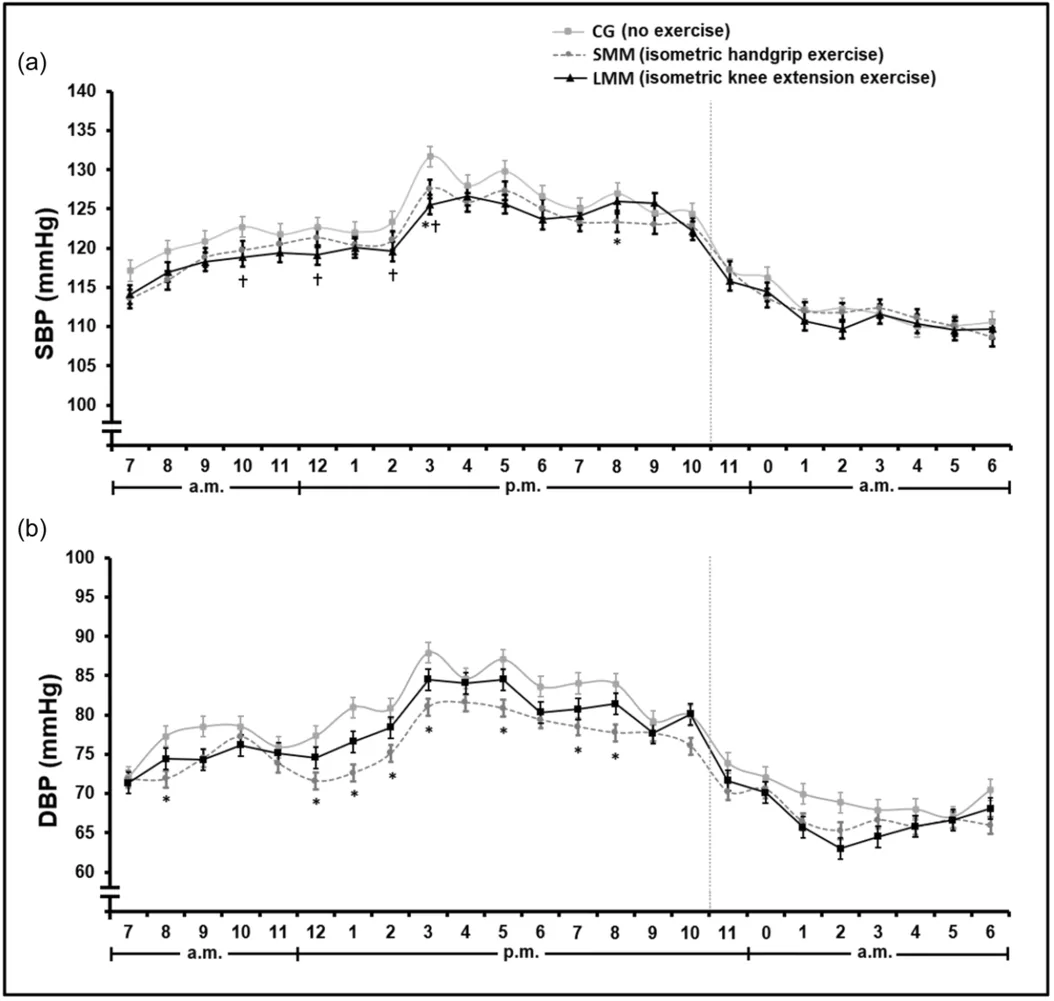
Differences between the intervention groups (small or large muscle mass) compared to the control group over 24‐h ambulatory blood pressure monitoring (ABPM). The chart shows hourly systolic blood pressure (SBP, Panel A) and diastolic blood pressure (DBP, Panel B) profiles in the CG control group (*n* = 22), SMM group (*n* = 22), small muscle mass (isometric handgrip exercise), and LMM group (*n* = 23), large muscle mass (isometric knee extension exercise). Values are presented as mean ± SD. Differences were tested using GEE, including group, time, and the group × time interaction, followed by Bonferroni‐adjusted pairwise comparisons when appropriate. *p < 0.05 SMM versus CG; ^†^ *p* < 0.05 LMM versus CG.

## 4 DISCUSSION

The present randomized clinical trial investigated the effects of 4 weeks of upper‐ and lower‐limb isometric training on ambulatory blood pressure in individuals with controlled hypertension. The main finding was that, after adjustment for baseline office blood pressure, significant between‐group differences were observed only for ambulatory blood pressure during the awake period, with lower systolic and diastolic blood pressure in the handgrip group compared with the control group. In contrast, no significant between‐group differences were observed for 24‐h or sleep ambulatory blood pressure, nor between the two exercise modalities. These findings indicate that the between‐group differences associated with short‐term isometric training were confined to the awake period, rather than being consistently observed across the entire 24‐h monitoring period or during sleep.

The 4‐week intervention period was selected to investigate short‐term blood pressure responses to isometric training. This duration is supported by previous investigations reporting blood pressure adaptations following 4 weeks of isometric exercise (Wright et al., 2026) and aerobic or resistance training (Collier et al., 2008). Although longer interventions are necessary to establish the persistence of these responses, 4 weeks represents a scientifically supported timeframe for investigating short‐term blood pressure adaptations. Thus, the present findings are consistent with previous investigations reporting modest reductions in ambulatory blood pressure following isometric exercise, although the available evidence remains heterogeneous. Taylor et al. (2019) demonstrated reductions in ambulatory blood pressure after training (Taylor et al., 2019). Conversely, studies employing handgrip exercise and ABPM have reported inconsistent findings, with some demonstrating significant reductions and others showing no meaningful changes (Farah et al., 2018; Goessler et al., 2018; Pagonas et al., 2017; Stiller‐Moldovan et al., 2012). More recently, a study comparing dynamic, isometric, and combined resistance training in treated hypertensive individuals further expanded the evidence regarding ambulatory blood pressure responses to different exercise modalities (Silva de Sousa et al., 2024). In addition, Palmeira et al. (2021) reported no significant changes in ambulatory blood pressure following 12 weeks of isometric handgrip training, despite improvements in office systolic blood pressure (Palmeira et al., 2021). Together, these findings suggest that ambulatory blood pressure responses to isometric exercise remain variable and may depend on factors such as participant characteristics, intervention duration, and training protocol.

Although the between‐group differences observed during the awake period were modest (approximately 2.4 mmHg for systolic and 4.5 mmHg for diastolic blood pressure), differences of this magnitude may be clinically relevant. Previous evidence indicates that reductions of only 2–5 mmHg in systolic blood pressure are associated with substantial reductions in cardiovascular risk, including a 14% reduction in stroke mortality, a 9% reduction in coronary heart disease mortality, and a 7% reduction in all‐cause mortality (Millar et al., 2014). Although these epidemiological estimates cannot be directly extrapolated to the present intervention, they illustrate the potential clinical relevance of relatively small differences in blood pressure.

A recent large‐scale meta‐analysis (Edwards, Jalaludeen, et al., 2023) comparing different exercise modalities in adults with normal blood pressure, pre‐hypertension, and hypertension demonstrated that aerobic, resistance, combined, high‐intensity interval, and isometric training all reduce blood pressure, with isometric exercise producing some of the largest reductions among hypertensive individuals. Nevertheless, considerable heterogeneity exists regarding the magnitude of these effects. Our findings are partially consistent with this evidence, as significant between‐group differences were observed for awake ambulatory blood pressure.

Most investigations of isometric exercise have employed handgrip protocols performed at approximately 30% of maximal voluntary contraction (Carlson et al., 2016; Farah et al., 2017; Farah et al., 2018; Gordon et al., 2018; Millar et al., 2013). In contrast, relatively few studies have evaluated isometric exercise involving larger muscle groups, such as knee extension (Baross, Brook, et al., 2022; Baross et al., 2013; Wiles et al., 2010), and even fewer have used ABPM as the primary outcome (Baross, Brook, et al., 2022; Baross et al., 2012; Taylor et al., 2019). Collectively, these studies demonstrate heterogeneous responses, reinforcing the need for additional randomized clinical trials using ABPM, which provides clinically relevant information beyond office blood pressure measurements.

One of the hypotheses of the present study was that greater muscle mass recruitment would produce greater antihypertensive adaptations because larger muscle groups generate greater cardiovascular stress during exercise (Lewis et al., 1985; MacDougall et al., 1985; Narloch & Brandstater, 1995). However, our results do not support this hypothesis. Although the lower‐limb training group consistently demonstrated numerically lower ambulatory blood pressure values than the control group, statistically significant differences were observed only for handgrip training during the awake period. Moreover, no significant differences were observed between the two exercise groups. These findings suggest that the ambulatory blood pressure response to short‐term isometric training is not determined solely by the amount of muscle mass recruited during exercise.

This observation differs somewhat from the conclusions of the meta‐analysis by Edwards et al. (2022), which suggested greater blood pressure reductions following exercises involving larger muscle groups, including wall squat and knee extension, than after handgrip training (Edwards et al., 2022). However, important methodological differences should be considered. Their meta‐analysis included studies involving participants with normal blood pressure, pre‐hypertension, and hypertension, with heterogeneous medication use and intervention durations ranging from four to twelve weeks. In contrast, our trial included only individuals with controlled hypertension receiving pharmacological treatment and employed a standardized 4‐week intervention. These differences in population characteristics and intervention duration may have contributed to the different pattern observed in the present study.

An interesting finding was that significant between‐group differences were confined to the awake period, whereas no significant differences were observed during sleep. This pattern may be related to differences in the physiological regulation of blood pressure across the 24‐h period. During wakefulness, blood pressure is strongly influenced by sympathetic activity, daily activities, and physical activity, whereas nocturnal blood pressure is more strongly influenced by circadian regulation and changes in autonomic activity. Thus, it is possible that the effects associated with short‐term isometric training are more readily detectable under daytime conditions. However, because the present study did not directly assess autonomic function or the mechanisms underlying daytime and nocturnal blood pressure regulation, this interpretation remains speculative.

The physiological mechanisms underlying the blood pressure response to isometric exercise remain incompletely understood. Current evidence suggests that reductions in peripheral vascular resistance resulting from improved endothelial function and vascular remodelling may contribute to chronic blood pressure reductions following isometric training (Edwards et al., 2022; Millar et al., 2014; Taylor et al., 2019). Alternative hypotheses include changes in cardiac output mediated by alterations in heart rate and stroke volume (Baross et al., 2012; Wiles et al., 2017). However, these mechanisms were not directly assessed in the present study and therefore cannot explain either the differences observed during the awake period or the similar ambulatory blood pressure responses between the two exercise modalities.

The present study has some limitations. Most importantly, baseline ABPM was not performed in the final study sample following a modification of the initially planned assessment schedule due to participant withdrawals during the pilot phase. Consequently, baseline comparability in ambulatory blood pressure could not be directly established, and within‐participant changes over the intervention period could not be determined. Although baseline office systolic and diastolic blood pressure measurements were included as covariates in the ANCOVA models, office blood pressure and ABPM represent distinct measurements. Therefore, statistical adjustment cannot fully address the absence of baseline ABPM, and the findings should be interpreted as adjusted between‐group differences in post‐intervention ambulatory blood pressure rather than changes from baseline. Another limitation is the absence of echocardiographic assessment of cardiac output and stroke volume, as well as direct measurements of peripheral vascular resistance or autonomic modulation, which could provide further insight into the physiological mechanisms responsible for the observed responses. Finally, awake and sleep periods were defined using fixed time intervals rather than individualized sleep diaries. Although this approach ensured consistent classification across participants, differences in individual sleep–wake schedules may have resulted in misclassification of some ambulatory blood pressure measurements, potentially influencing the observed awake and sleep blood pressure estimates.

In conclusion, after 4 weeks of isometric training, significant between‐group differences in ambulatory blood pressure were observed only during the awake period. After adjustment for baseline office blood pressure, the handgrip group exhibited lower awake systolic and diastolic blood pressure than the control group, whereas no significant differences were detected for 24‐h or sleep blood pressure. Lower‐limb isometric training showed a similar numerical pattern but did not differ from the control group, and no significant differences were observed between the two exercise modalities. These findings support further investigation of isometric exercise as an adjunctive strategy for blood pressure management and suggest that greater muscle mass recruitment does not necessarily result in greater ambulatory blood pressure effects. These findings should be considered preliminary and warrant confirmation in longer randomized clinical trials incorporating both baseline and post‐intervention ABPM assessments.

## AUTHOR CONTRIBUTIONS

**Patrícia C. Oliveira:** Conceptualization; methodology; data acquisition; writing—original draft. **Gustavo Waclawovsky:** Conceptualization; formal analysis; interpretation of data; writing—original draft; writing—review and editing. **Alexandre M. Lehnen:** Conceptualization; methodology; formal analysis; interpretation of data; writing—review and editing; final approval of the manuscript for submission.

## CONFLICT OF INTEREST STATEMENT

The authors declare no conflicts of interest. They all have read and approved the final manuscript. Also, the authors acknowledge the use of ChatGPT (OpenAI, San Francisco, CA, USA) to assist with English language editing. All scientific content, data interpretation, and conclusions remain the sole responsibility of the authors. The dataset was made available as a supplementary file.

## COMPLIANCE WITH ETHICAL STANDARDS

This was a randomized, parallel‐group, assessor‐blinded clinical trial conducted in accordance with the CONSORT Statement and the Declaration of Helsinki, and registered on ClinicalTrials.gov, NCT07147595. The datasets will be made available as supplementary material.

## Data Availability

The data that support the findings of this study are available in the supplementary material of this article.
